# Investigating the Accessibility of Voice Assistants With Impaired Users: Mixed Methods Study

**DOI:** 10.2196/18431

**Published:** 2020-09-25

**Authors:** Fabio Masina, Valeria Orso, Patrik Pluchino, Giulia Dainese, Stefania Volpato, Cristian Nelini, Daniela Mapelli, Anna Spagnolli, Luciano Gamberini

**Affiliations:** 1 Human Inspired Technologies Research Center University of Padova Padova Italy; 2 Department of General Psychology University of Padova Padova Italy; 3 L'Incontro Social Enterprise Castelfranco Veneto Italy; 4 Day Center for Severe Acquired Brain Injury Opere Pie—Istituto Pubblico di Assistenza e Beneficienza Pederobba Italy

**Keywords:** voice assistants, accessibility, cognitive functions, disability, ambient assisted living

## Abstract

**Background:**

Voice assistants allow users to control appliances and functions of a smart home by simply uttering a few words. Such systems hold the potential to significantly help users with motor and cognitive disabilities who currently depend on their caregiver even for basic needs (eg, opening a door). The research on voice assistants is mainly dedicated to able-bodied users, and studies evaluating the accessibility of such systems are still sparse and fail to account for the participants’ actual motor, linguistic, and cognitive abilities.

**Objective:**

The aim of this work is to investigate whether cognitive and/or linguistic functions could predict user performance in operating an off-the-shelf voice assistant (Google Home).

**Methods:**

A group of users with disabilities (n=16) was invited to a living laboratory and asked to interact with the system. Besides collecting data on their performance and experience with the system, their cognitive and linguistic skills were assessed using standardized inventories. The identification of predictors (cognitive and/or linguistic) capable of accounting for an efficient interaction with the voice assistant was investigated by performing multiple linear regression models. The best model was identified by adopting a selection strategy based on the Akaike information criterion (AIC).

**Results:**

For users with disabilities, the effectiveness of interacting with a voice assistant is predicted by the Mini-Mental State Examination (MMSE) and the Robertson Dysarthria Profile (specifically, the ability to repeat sentences), as the best model shows (AIC=130.11).

**Conclusions:**

Users with motor, linguistic, and cognitive impairments can effectively interact with voice assistants, given specific levels of residual cognitive and linguistic skills. More specifically, our paper advances practical indicators to predict the level of accessibility of speech-based interactive systems. Finally, accessibility design guidelines are introduced based on the performance results observed in users with disabilities.

## Introduction

### Background

Voice-activated technologies are becoming pervasive in our everyday life [1,2]. In 2017, 46% of Americans reported using voice-activated technologies [3-5]. One of the most prominent application domains is the domestic environment, where voice assistants, a branch of voice-activated technologies, allow the user to control and interact with several home appliances in a natural way by uttering voice commands [6,7]. When integrated into a smart house, voice assistants allow the user to perform numerous everyday actions without the need to move and reach the actual object. More specifically, the user can operate all the devices that are connected, ranging from switching the lights on and off to opening and closing the doors and windows, for instance.

Research on voice assistants is focused mainly on the general population. Indeed, the studies investigating user experience and usability of voice assistants mainly involved able-bodied users [3,8-11], thereby neglecting a broad community of users with disabilities. However, people suffering from motor and cognitive impairments would significantly benefit from the possibility of controlling home appliances and personal devices remotely. Voice assistants hold the potential to enable individuals with disabilities to govern their houses without the need to constantly depend on caregivers [3,12].

One of the obvious barriers that some users with disabilities can encounter by interacting with voice assistants is related to speech impairments [13] that are a frequent secondary consequence of motor disorders [14]. Although most voice assistants exploit machine learning algorithms to adapt to the user and increase their speech recognition accuracy over time [15,16], these systems are still designed and developed for people with clear and intelligible speech. Thus, the difficulty of clearly utter sentences and speaking with adequate vocal intensity may represent a relevant accessibility challenge of voice assistants. The accessibility of voice assistants has not been thoroughly investigated yet. In this study, we explored how users with motor, linguistic, and cognitive disabilities interact with a commercial voice assistant in a natural situation. More specifically, the aim was to investigate the role of cognitive and linguistic functions to predict the performance of individuals affected by physical, linguistic, and cognitive difficulties in interacting with a voice assistant.

### Voice Assistants for Users With Disabilities

Studies investigating the interaction between users with disabilities and voice assistants are still sparse. However, some evidence is starting to shed light in this field. Recently, Pradhan and colleagues [7] investigated the opinions of disabled users who regularly deploy voice assistants by examining their reviews. Most comments (about 86%) were positive, highlighting how the device has made it easier to accomplish specific tasks autonomously (eg, playing songs). The complaints were mainly focused on the lack of desired features, yet users pointed out that the main challenges they have in interacting with the voice assistant were due to the need to speak aloud and respect a precise timing for uttering the command. On the whole, these findings were confirmed by a following interview-based study with users with disability [7].

Ballati and colleagues [17] investigated to what extent people affected by speech impairments could be understood by three different voice assistants available off the shelf. More specifically, accuracy in speech processing was tested using sentences extracted from the TORGO database, which includes the recordings of 8 English speakers with dysarthria [18]. The sentences extracted were spoken to the voice assistants, and the accuracy across systems was compared. Each system processed the sentences one by one, while the experimenter scored the system accuracy with respect to the ability of the system to understand the sentence and consistency of the answer by the system. Results of this study revealed a general speech recognition accuracy of 50% to 60%, with all three systems having similar performance. These findings were partially confirmed with dysarthric Italian patients [19], where authors found different performance accuracy across the voice assistants.

While insightful, the studies reported above have limitations that might make it challenging to generalize the results. First, the actual speech abilities of the users were not assessed because they were either self-reported [7] or not reported at all [17,19]. This approach fails to provide clear indications for the design of voice assistants, as it does not highlight the users’ needs. In addition, previous studies focused on speech abilities, neglecting cognitive skills. Cognitive skills were proven to affect the ability to operate a voice-controlled device by Weiner and colleagues [20]. In this study, the voice-controlled system showed a decrease in accuracy of speech recognition when the speakers suffered from Alzheimer disease or age-related cognitive decline. Furthermore, patients with Alzheimer disease experience difficulties interacting with a voice-controlled robot because of the timing imposed by the device [21].

Some of the previous research [17,19] did not even involve humans as participants, as they relied on prerecorded sentences. While this ensures high reliability in terms of assessing the robustness of the system, it also fails to account for the variability of individual performances and motivation behind the actual use of the device. Likewise, Pradhan and colleagues [7] found that about a third of the reviews analyzed were written by caregivers, who may have reported their viewpoint, misleading the perspective of the person they assisted. Finally, to the best of the writers’ knowledge, the research available so far was conducted in laboratory settings, where the background noise is controlled, if any, and where there are no group interactions, as is likely to happen in a household.

### Speech and Cognitive Factors Accounting for User Performance

Speech and cognitive skills play a significant role in the ability to effectively control voice assistants [17,19,22]. To properly convey a voice command, users must adequately control the speed and rhythm of speech. As reported in a previous study, speech disfluency can represent an accessibility barrier to voice assistants. For instance, long hesitations or pauses can be misinterpreted by the system as a sentence delimiter [23], causing an alteration of speech segmentation. Moreover, users must be able to correctly articulate words, especially multisyllable words (eg, temperature) or specific words that may require more effort to be articulated [24]. A further aspect to properly interact with these devices is the voice intensity, which should be sufficiently loud to make voice assistants detect and segment the sounds [7].

Along with these speech skills, cognitive abilities are required to utter a command. The user must remember specific keywords and specific sequences of words to operate the system. These abilities involve memory functions, specifically long-term memory and working memory, both crucial when interacting with voice interfaces [25]. In addition, the user must respect a specific timing to provide the commands, a capacity that counts on executive functions, namely a set of functions needed to plan and control actions [26]. Not least, to properly use a voice assistant, the user must also monitor the feedback of the system (which sometimes consists of simple lights) and correctly interpret it. Such skills rely on underlying attention processes.

## Methods

### Study Design

This study was meant to assess the accessibility of a commercial voice assistant. In particular, we investigated whether specific cognitive and/or linguistic skills were related to the effectiveness of the interaction. To this end, the study consisted of two phases. In phase 1, participants were involved in group sessions, in which they were invited to interact with the voice assistant by performing several realistic tasks in a living laboratory (eg, switching on the light). Each group session involved 4 participants. This choice was motivated by our desire to build a friendly and informal setting that could facilitate interaction and prevent the feeling of being in a testing situation. Group sessions were video recorded to allow offline analysis of participant performances. In phase 2, participants received an evaluation of their neuropsychological and linguistic functions. The two phases of the study took place in different settings and on separate days and required different experimental materials. The study was approved by the Ethics Committee of the Human Inspired Technologies Research Center, University of Padova, Italy (reference number 2019_39).

### Participants

A total of 16 participants (9 males, 7 females) took part in the study. The mean age of the sample was 38.3 (SD 8.6) years (range 22 to 51 years). On average, they had 11.8 (SD 2.7) years of education (range 8 to 18 years). To partake in the study, participants had to meet the following inclusion criteria: (1) suffering from ascertained motor impairments and related language difficulties and (2) needing daily assistance from at least one caregiver. The sample was characterized by 6 participants affected by congenital disorders, 2 participants with neurodegenerative disorders, 4 participants affected by traumatic brain injury, and 4 participants with nontraumatic brain injury (ie, tumor). The heterogeneity of the sample well represents the population that can be found in daycare centers. Participants were indeed recruited from a daycare center for people with disabilities, with which the research team collaborates. Before enrollment, all invited participants received an explanation of the activity. Upon agreement, they were provided written informed consent (if necessary, the individual’s legal guardian was informed about the scope and unfolding of the activity and gave the informed consent for the person they assisted to partake in the study). In any case, informed consent was given prior to their enrollment. Participants received no compensation for taking part in the study.

### Phase 1: Interaction With the Voice Assistant

#### Setting

**Figure 1:**
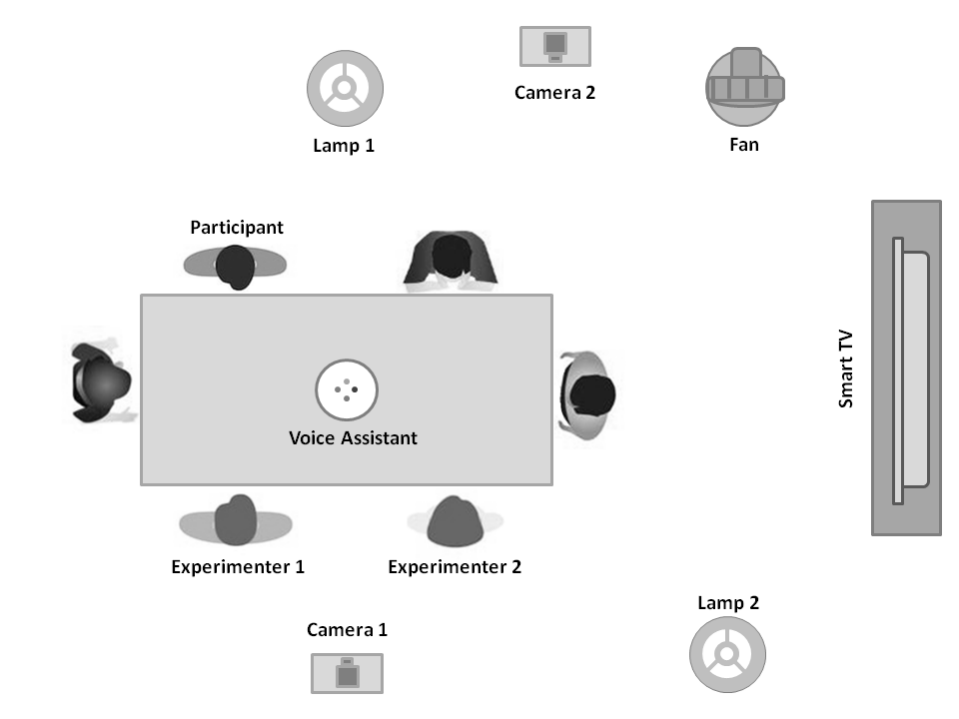
Representation of the experimental setting.

The first phase took place in a living laboratory. The room was furnished to resemble a living room with a large table in the middle. The voice assistant was placed at the center of the table, around which participants and experimenters were sitting (Figure 1). The laboratory was equipped with several devices that were connected to the voice assistant and could be controlled by prompting voice commands. All of the voice-controlled devices were placed so that users could easily see them. The room was also equipped with two camcorders to video record the sessions. One camera was placed above the table and enabled the observation of users’ interactions with the voice assistant. The other camera served to record the outcomes of the interaction (Figure 1).

#### Equipment

For this study, a commercial voice assistant was deployed. More specifically, we chose to use Google Home (Google LLC), given its growing popularity. Two lamps and a floor fan were connected to smart plugs, which were in turn connected to the voice assistant, thereby enabling control of the switch on/off and light color change (for the lamps only). A 50-inch television was connected to Chromecast (Google LLC), which was in turn connected to Google Home. By doing this, it was possible to operate the TV using voice commands. For the video recordings, two video cameras were installed, one was a C920 Pro HD (Logitech) and the other one was a Handycam HDR-XR155E (Sony Europe BV).

#### Tasks

Participants were invited to individually prompt some commands to the voice assistant, as indicated by the experimenter. The tasks comprised turning on/off the fan and the lights, changing the color of the light, interacting with the TV (activating YouTube, Spotify, and Netflix), and making specific requests to the voice assistant (eg, “set an alarm for 1 pm”). The full list of commands that participants were asked to speak can be seen in Textbox 1.

**The list of voice commands that participants were asked to speak during the first phase of the study.**

**Fans**

- Turning on/off

**Lamps**

- Turning on/off
- Changing colors
- Changing light intensity

**TV (YouTube)**

- Selecting videos
- Increasing/decreasing volume

**TV (Netflix)**

- Selecting movies
- Pausing movies
- Playing movies

**TV (Spotify)**

- Selecting songs
- Increasing/decreasing volume

**Voice assistant**

- Asking for the latest news
- Asking for the weather forecast
- Setting an alarm

#### Procedure

Participants were first welcomed in the living laboratory and invited to make themselves comfortable. They were reminded about the aim and the unfolding of the activity. In addition, they were shown the camcorders and after they all proved to be aware of them, the video recording started. At this point, the experimenter showed how the voice assistant worked by prompting some example commands and properly explained the correct sequence of words to convey the command. Next, participants were allowed to familiarize themselves with the voice assistant until they felt confident. When they considered themselves ready, the experimental session started. The experimenter asked each participant to individually perform the selected tasks (Textbox 1). The tasks were not proposed in a strict order across participants. To keep the session lively and prevent boredom and fatigue, the tasks were alternated across participants. Should a participant fail to accomplish a requested task (eg, the voice assistant did not respond in the expected manner), the experimenter gently encouraged them to try again. A fixed number of attempts was not set a priori to prevent participants from feeling frustrated as a consequence of repeated failed attempts. Participants were allowed to try until they felt comfortable.

Once the task list was completed by all participants, the experimenter asked them their impressions about the voice assistant in a semistructured group interview. The questions regarded an overall evaluation of the pleasantness of the voice assistant (from 1 to 10), in which rooms it would be more helpful, if they would like to have it in their own houses, and which additional functions they would like to control. Phase 1 took about 2.5 hours.

### Phase 2: Neuropsychological and Linguistic Assessment

#### Data Collection

All of the participants involved in phase 1 received an individual examination by a trained neuropsychologist and a speech therapist, who were both blind to the outcomes of the users’ performances with the voice assistant. Several assessment tools were selected and adopted. More specifically, the neuropsychological functions were assessed with the Addenbrooke’s Cognitive Examination–Revised (ACE-R) [27] and the Frontal Assessment Battery (FAB) [28]. The linguistic assessment was conducted by collecting several measures, namely participant vocal intensity, and other speech production indices gathered using the standardized Italian version of the Robertson Dysarthria Profile [29]. The evaluation sessions took place in a quiet room at the daycare center where participants were recruited and lasted about 1.5 hours for the neuropsychological evaluation and 2 hours for the linguistic evaluation.

#### Neuropsychological and Linguistic Tests

The ACE-R [27] is a screening test originally proposed as an extension of the Mini-Mental State Examination (MMSE) [30]. The ACE-R allows the evaluation of 5 cognitive domains, attention/orientation, memory, verbal/category fluency, language, and visuospatial ability, in addition to providing the MMSE score. Attention/orientation is assessed by asking the participant about the date, season, and current location where the evaluation is taking place, as well as repeating 3 single words and doing serial subtractions. Memory consists of items that evaluate episodic and semantic memory. Verbal and category fluency require the ability to list in 1 minute as many words as possible complying with a verbal criterion and a category criterion. Language includes several subtasks, requiring speech comprehension, naming figures, repeating words and sentences, reading regular and irregular words, and writing. Finally, visuospatial ability consists of copying and drawing specific pictures.

With respect to the MMSE, it represents a general index of cognitive functioning ranging from 0 to 30. A score below 24 may indicate the presence of cognitive impairment [30].

##### Frontal Assessment Battery

The FAB [28] is a brief inventory for the evaluation of executive functions. It is composed by 6 subscales exploring domains: conceptualization (similarities test), mental flexibility (verbal fluency test), motor programming (Luria motor sequences), sensitivity to interference (conflicting instructions), inhibitory control (go/no-go test), and environmental autonomy (prehension behavior). Each domain consists of 3 items and is scored from 0 (unable to complete the requests) to 3 (fully able to fulfill the requests). The maximum overall score for the FAB is 18.

##### Vocal Intensity

Vocal intensity reflects the loudness of the voice. Physically, it represents the magnitude of the oscillations of the vocal folds, and it is measured in decibels (dB). In this study, vocal intensity was collected by using the PRAAT software [31], a tool for speech analysis. Participants were invited to repeat aloud a specific sentence (ie, “Turn off the light, turn on the TV” in their native language) for 5 minutes at a distance of 1.5 meters from the recording device.

##### Speech Production

An expert speech and language therapist assessed participant speech production. The protocol adopted for the evaluation was extracted from the Robertson Dysarthria Profile [29]. This test is divided into 8 subscales (ie, intelligibility, respiration, phonation, facial muscles, diadochokinesis, oral reflexes, prosody, articulation), each including several items. The therapist assigns a score on a 4-point scale (1 = severe, 2 = moderate, 3 = mild, 4 = normal) for each item of the test. In this study, the subscales considered were prosody and articulation. More specifically, for prosody (2 items) the items assessed the speed and rhythm of speech production. With regard to articulation (5 items), the items considered the ability to articulate single letters (consonants and vowels) and clustered letters (groups of consonants and multisyllable words), as well as the capacity to repeat sentences.

### Data Analysis

The data analysis comprised analysis of the video recordings to assess the extent to which users were capable to effectively interacting with the voice assistant. The outcomes of the analysis were summarized into a performance index. The index was then associated with the neuropsychological and linguistic measures collected in the second phase of this study. Since the main purpose of this study was the identification of predictors (cognitive and/or linguistic) capable of accounting for an effective interaction with the voice assistant, multiple linear regression models were run.

### Video Analysis

The two video streams recorded during the sessions were synchronized into a single video file using a video editing software. The resulting video was then imported into a dedicated software for the analysis (The Observer XT 12, Noldus Information Technology Inc). The analysis was conducted in two passes. During the first pass, two of the authors watched the videos and selected the events of interest: the experimenter’s requests, participants’ actions, and voice assistant’s responses. The two researchers then agreed on the events to code, defining the objective triggers detailing the beginning and the end of each. A trained coder was in charge of rating the videos.

For each participant, the number of attempts they made for each task request and the resulting outcome were coded. More specifically, the beginning of an attempt was coded when the experimenter prompted the participant to try to accomplish a given task. The attempt ended with either the actual activation of the intended function (successful outcome) or with a failure to observe the expected outcome (unsuccessful outcome). In particular, unsuccessful outcomes were further categorized based on the type of error made by the participants. Four categories of errors were identified:

- Timing errors included all of the unsuccessful outcomes caused by the participant not respecting the timing imposed by the system (eg, the participant uttered the waking command “Hey Google” and did not wait for the system to reply before prompting the full command)
- Phrasing errors comprised all the failed attempts that followed an incorrect sequence of words to prompt the command (eg, the participant saying “Hey Google...put the red the lamp” instead of “Hey Google...make the lamp red”)
- Comprehension errors referred to all mistakes participants made because they could not understand the experimenter’s request (eg, changing the color of the lamp instead of turning it off)
- Pronunciation errors included all of the failures that followed a wrong articulation of one or more words within the sentence (eg, participants struggling to pronounce words that were not in their native language, such as Netflix)

Participants’ attempts could also be coded as self-corrections (with successful or unsuccessful outcome) when the participant realized autonomously that the command was wrong and tried to amend it.

To understand whether participants were able to prompt commands to the voice assistant, an overall performance index was computed expressing the percentage of successful attempts and the total number of attempts. Importantly, self-corrections with successful outcomes were considered successful attempts whereas self-corrections with unsuccessful outcomes were considered unsuccessful attempts.

### Neuropsychological and Linguistic Assessment

Regarding the neuropsychological measures, not all participants were able to complete all of the subscales of the ACE-R. More specifically, several participants could not fully complete some items of the ACE-R (eg, drawing a clock) because of their physical impairments (eg, dystonia). However, since all participants could complete at least the items of the MMSE, only the MMSE score was considered in the multiple linear regression models, in addition to the FAB score. With regard to the linguistic assessments, all the collected measures were considered in the regression models.

### Multiple Linear Regression Models

Data were statistically analyzed using RStudio software version 1.2 (RStudio PBC). To investigate which predictors of the performance index (participant performances during the use of the voice assistant) are best, multiple linear regression models were adopted. In order to make accurate predictions, we considered, among several models, the one that best described the data. The best model was identified by adopting a selection strategy based on the Akaike information criterion (AIC). The AIC value provides an estimation of the quality of a model given several other candidate models. The AIC considers both the complexity of a model and its goodness of fit. According to the AIC, given a set of models, the one characterized by the lowest AIC is the best [32].

The neuropsychological and linguistic predictors entered in the models were the MMSE score, FAB score, vocal intensity (dB), and scores obtained from the 2 items of the prosody subscale and 5 items of the articulation subscale of the Robertson Dysarthria Profile. More specifically, the linear regression models were performed entering the predictors grouped into four clusters: (1) neuropsychological cluster (ie, MMSE and FAB), (2) vocal intensity cluster (ie, dB), (3) prosody cluster (ie, speed and rhythm), and (4) articulation cluster (ie, initial consonants, vowels, groups of consonants, multisyllable words, and repetition of sentences). The latter two clusters consisted of the items in the Robertson Dysarthria Profile. Since the forced entry method was adopted, the order in which predictors were entered in the model did not affect the results.

## Results

### Video Analysis

The performance index extracted from the video analysis shows that participant accuracy was on average 58.5% (SD 18.6%). The most frequent type of errors made by participants were phrasing errors (75/182, 41.2%). Participants mainly had problems uttering long commands, especially when they were required to respect a specific syntax. It should be noted that uttering the right sequences of words was not problematic to the same extent for all participants, as one participant never made this type of error, while one made it 21 times.

Timing errors were the second most frequent type of error (74/182, 40.7%), and they can be clustered into anticipatory timing errors and delayed timing errors. More specifically, as for the anticipatory timing errors, participants tended not to wait for the system to reply to the waking command before prompting the actual command. For one participant, respecting the timing seemed particularly difficult, as they made this type of error 30 times. To a lesser extent, with regard to the delayed timing errors, participants waited too long after the system had replied to the waking command. In many cases, the actual command overlapped to the system prompting the error message “Sorry, I don’t know how to help you.”

Less frequent were the comprehension errors (19/182, 10.4%) and pronunciation errors (14/182, 7.7%). Regarding the former, participants mainly tended to misunderstand the most complex commands (eg, playing a video on YouTube). Regarding the latter, users had some difficulties with English words, like Netflix. Nevertheless, the system could successfully respond even when they had strong dialectal stress.

Overall, all participants enjoyed the interaction with the voice assistant. Indeed, the general evaluation of the system was extremely positive, with a mean score of 9.4 (SD 1.2). As for the rooms in which participants would like to install the voice assistant, 8 of them suggested the bedroom and 4 the kitchen. On the whole, all participants would like to have a voice assistant at their own house. Finally, with regard to the functions that participants would have liked to implement in their own house, they mentioned playing music (n=5) and controlling the home automation (n=5), such as opening/closing windows and doors.

Interestingly, during the interaction with the voice assistant, several participants provided their spontaneous opinions highlighting the benefits and drawbacks of the system. For instance, P3 stated: “Since my shoulder hurts, it is useful because it is easier when I have to open doors.” However, P3 claimed as well: “sometimes it does not understand me and I am afraid to crash the Google program.” Another participant mentioned some difficulties as well, especially concerning the general utility of having a voice assistant at home. P9 stated: “I cannot think as before [the accident], it is not so easy to have such a device at home, it might not be useful.”

### Neuropsychological and Linguistic Assessment

Table 1 shows the raw scores from participants in the neuropsychological and linguistic assessments made in the second phase of the study. With regard to the neuropsychological scores, participants showed a mean MMSE score of 26.1 and a mean FAB score of 12.6. Concerning the linguistic assessment, participants had a mean vocal intensity of 61.6 dB. Finally, the mean scores of the Robertson Dysarthria Profile indicated speed of speech of 2.7, and rhythm of speech of 2.6. Overall, these scores indicated mild to moderate prosody difficulties. Finally, the mean scores of items measuring articulation abilities showed mild issues regarding the pronunciation of initial consonants (mean 3.3), vowels (mean 3.3), groups of consonants (mean 3.2), multisyllable words (mean 3.3), and the repetition of sentences (mean 3.1).

**Table 1:** Summary of participant scores from the neuropsychological and linguistic assessments.

Measure		Mean score (SD)
Mini-Mental State Examination		26.1 (2.9)
Frontal Assessment Battery		12.6 (3.8)
Vocal intensity (dB)		61.6 (4.2)
**Prosody**		
	Speed of speech production	2.7 (0.7)
	Rhythm of speech production	2.6 (0.7)
**Articulation**		
	Initial consonants	3.3 (0.6)
	Vowels	3.3 (0.5)
	Groups of consonants	3.2 (0.7)
	Multisyllable words	3.3 (0.6)
	Repetition of sentences	3.1 (0.6)

### Multiple Linear Regression Models

In order to identify the best model to predict participant accuracy (assessed as the performance index), several multiple linear regression models were considered. Multimedia Appendix 1 shows all estimated models with their respective AIC scores. Comparing the AICs in all the models, model *ad* (F_6,9_=4.91, *P*=.02, *R*^2^=.77), which included the neuropsychological and articulation clusters, was the best one (AIC 130.69; Multimedia Appendix 1).

When checking for the coefficients of this model, 2 predictors were found to explain a significant amount of the variance of accuracy. The predictors that significantly accounted for accuracy were the MMSE (β=6.16, *t*_9_=3.88, *P*=.004) and repetition of sentences (β=31.14, *t*_9_=2.71, *P*=.02). Of importance, among the nonsignificant predictors, 3 (ie, initial consonant, group of consonants, and multisyllable words) had a variance inflation factor (VIF) >10 (tolerance statistics: 1/VIF<0.1), showing multicollinearity [33,34]. As a consequence, a new model was performed, removing all the nonsignificant and collinear predictors by entering only the MMSE and repetition of sentences. The results confirmed the previous model, namely that the MMSE and repetition of sentences were significant predictors of accuracy: MMSE (β=3.70, t_13_=3.26, *P*=.006) and repetition of sentences (β=22.06, t_13_=4.16, *P*=.001). The AIC value of this final model was 130.11, showing that it was the best model compared with the previous ones (Multimedia Appendix 1).

To test the assumptions of the linear regression model, diagnostic statistics were performed. The model met the assumption of independence (Durbin-Watson 2.29, *P*=.68). The Q-Q plot of standardized residuals suggested that the residuals were normally distributed. Tolerance statistics (1/VIF) indicated that multicollinearity was not a concern (MMSE tolerance .92; repetition of sentences tolerance .92).

The standardized values were .57 (MMSE) and .73 (repetition of sentences). The first value suggests that as the MMSE increases by 1 standard deviation (2.89 points), the performance index increases by 1 standard deviation as well (10.6%). This prediction is true only if the repetition of sentences is constant. The second standardized value predicts that every time the repetition of sentences improves by 1 standard deviation (0.6 points), the performance index increases of 1 standard deviation (13.6%). This interpretation is true only if the MMSE is fixed.

## Discussion

### Principal Findings

This work aimed to investigate whether cognitive and/or linguistic functions could predict the user’s performance in operating an off-the-shelf voice assistant. To this end, a group of users suffering from motor and cognitive difficulties was invited to a living laboratory. The lab was purposefully equipped with a voice assistant connected to several smart devices (ie, TV, lamps, floor fan), and participants were asked to perform specific tasks following the experimenter’s instructions. In order to assess user performances, interactions with the voice assistant were video recorded. Cognitive and linguistic functions were assessed with standardized inventories and subsequently related to the user performances with the voice assistant.

The performance index was found to be predicted by the overall cognitive abilities, as assessed by the score on the MMSE and by the ability to repeat sentences. In other words, a minimum level of residual cognitive functioning (ie, MMSE score above the cutoff [≥24]) is recommended to effectively operate a voice assistant. Among the linguistic skills, the ability to repeat sentences was necessary. These findings contribute to provide specific indications of the level of inclusion of commercial voice assistants.

More generally, the average accuracy was around 60%, extending previous findings that were limited to synthesized utterances [17]. Different than the previous studies, we arranged a living lab and involved actual users with disabilities in a realistic group situation. This approach allowed us to identify and categorize the most prominent types of errors emerging during the interaction. The most frequent mistakes were phrasing errors (41.2%), highlighting the difficulties of participants to respect the syntax of the voice command, especially when the command was long. The second most frequent error consisted of the difficulty in respecting the timing imposed by the device (40.7%), as already reported by Pradhan and colleagues [7]. Specifically, participants uttered the command too quickly or too slowly, showing a tendency to ignore the feedback of the voice assistant. This was probably due to the lack of saliency of the feedback provided by Google Home after the waking command [35], which consists only of dim lights moving on top of the device. Additionally, two other types of errors emerged, relating to difficulty of comprehension of the request (10.4%) and pronunciation issues (7.7%). The latter was limited to English words. These findings suggest significant implications for the design of universal voice assistants. First, more salient feedback should be included to make it easier for users with disabilities to interact with the system. Additionally, the timing should be adjustable to better respond to the actual abilities of the user and adapt to their proficiency in using it over time. Finally, to increase the likelihood of users remembering how to operate the voice assistant, commands should include familiar words.

These results are particularly relevant because they provide new implications for the design of voice assistants using an inclusive design perspective that also considers users with special needs. On the other hand, these findings can provide an indication to caregivers, both family members and health care professionals, for choosing assistant technologies that are suitable for the people they assist. More specifically, the ability to interact and use voice assistants does not depend exclusively on linguistic skills, as it could seem. In fact, aspects related to cognitive functions, in particular the global level of cognitive functioning, seem to play a crucial role. Hence, linguistic and cognitive abilities predict performance with voice assistants. Users with severe cognitive impairment (MMSE score <18) [36] may still be able to use these systems effectively (performance index >50%) if their level of linguistic skills is normal (Robertson Dysarthria Profile = 4) [29], which somehow compensates for the cognitive difficulties. Similarly, a user with severe difficulty in articulating sentences may successfully use voice assistants if they have a normal level of cognitive functioning such that they can invoke compensative strategies. Taken together, these findings may serve as promising indicators to foresee the degree of accessibility of voice assistants. Importantly, the predictors employed in this study are extracted from standardized inventories that are highly widespread and administered in many clinical environments.

Finally, despite the mistakes, participants positively received the system and enjoyed their experience, consistent with the findings of Pradhan and colleagues [7]. Users found the system useful and reported that they would like to have it at their own houses. In addition, they suggested that such a system would be helpful in compensating for their difficulties with movements (eg, opening doors). The positive user opinions about the system revealed the general acceptance of voice assistants, highlighting the importance of using these mainstream systems in the field of assistive technologies in order to help users with disabilities regain some independence and increase their quality of life.

This study suggests that with specific and targeted adjustments a commercial voice assistant can be turned into an assistive technology that can effectively complement the individual’s skills. Indeed, voice assistants could offer tremendous benefits. First of all, these systems are widespread and inexpensive compared with assistive technologies, which are often harder to find and costly. Furthermore, assistive technologies can be stigmatizing. The fear of feeling exposed and feelings of autonomy and dignity loss are significant barriers to the adoption of assistive technology [37]. On the other hand, the popularity of voice assistants, as well as their appealing design, may make them really inclusive technology, being helpful to individuals with or without disabilities.

### Limitations

We acknowledge that this study has some limitations. First, the sample size was limited to 16 participants. Therefore, further studies should extend our findings with larger and even more heterogenous samples. In addition, we have explored a likely use scenario, where users interact with the voice assistant in a group situation, as happens in shared living environments. Nevertheless, future experiments should also investigate a use scenario in which the user operates the system individually to examine more closely the interaction between the individual and the voice assistant.

### Conclusions

In this work we report on a group experiment involving users with motor, linguistic, and cognitive difficulties that was meant to predict participant performances based on their level of cognitive and linguistic skills. Previous studies did not involve actual users or consider their capabilities. For the first time, we conducted an experiment in a living lab with individuals with disabilities and provide a detailed report of their performances and difficulties. More importantly, participant performances showed they could be predicted by their residual level of cognitive and linguistic capabilities. In addition, these results contribute to the field of assistive technology by describing the different types of errors made by users and providing design implications.

The enthusiastic reaction of participants highlights the potential of voice assistants to provide or return some autonomy in basic activities, like turning the light on/off when they are lying in bed. Further research effort should be devoted to fine-tuning voice assistants to better serve users’ needs and evaluating in the field to what extent the systems are actually helpful. To conclude, by polishing the existing widespread voice assistants, there will be the concrete opportunity to increase the quality of life of people with disabilities by providing them with truly inclusive technology.

## Appendix

Multimedia Appendix 1 Linear regression models considered in the analyses with their respective values of R2, adjusted R2, and Akaike information criterion.

## Abbreviations

ACE-R: Addenbrooke’s Cognitive Examination–Revised
AIC: Akaike information criterion
dB: decibel
FAB: Frontal Assessment Battery
MMSE: Mini-Mental State Examination
VIF: variance inflation factor
