# Supplementary material for: Investigating the Accessibility of Voice Assistants With Impaired Users: Mixed Methods Study
Source: J Med Internet Res. 2020 Sep 25;22(9):e18431. doi: 10.2196/18431 (PMC7547392; doi:10.2196/18431)
Supplement: Multimedia Appendix 1 [file jmir_v22i9e18431_app1.docx]

Multimedia Appendix 1. The list of the linear regression models considered in the analyses with their respective values of *R*^2^, adjusted *R*^2^ (Adj. *R*^2^), and AICs. Predictors entered in each model are indicated by the symbol “+”. Letters a, b, c, and d refer to predictor clusters, respectively: (a) neuropsychological cluster; (b) vocal intensity cluster; (c) prosody cluster; and (d) articulation cluster.

| Model |  | | | | | | | | | | *R*^2^ | Adj. *R*^2^ | AIC |
| --- | --- | --- | --- | --- | --- | --- | --- | --- | --- | --- | --- | --- | --- |
|  | cluster  a | | cluster  b | cluster  c | | cluster  d | | | | |  |  |  |
|  | MMSE | FAB | dB | Speed | Rhythm | Initial Consonants | Vowels | Groups of consonants | Multi-syllable words | Repetition of sentences |  |  |  |
| abcd | + | + | + | + | + | + | + | + | + | + | 0.80 | 0.5 | 134.11 |
| abc | + | + | + | + | + |  |  |  |  |  | 0.43 | 0.15 | 142.83 |
| abd | + | + | + |  |  | + | + | + | + | + | 0.80 | 0.62 | 130.79 |
| acd | + | + |  | + | + | + | + | + | + | + | 0.77 | 0.51 | 134.42 |
| bcd |  |  | + | + | + | + | + | + | + | + | 0.45 | -0.04 | 146.47 |
| ab | + | + | + |  |  |  |  |  |  |  | 0.21 | 0.01 | 144.19 |
| ac | + | + |  | + | + |  |  |  |  |  | 0.43 | 0.22 | 141.07 |
| ad | + | + |  |  |  | + | + | + | + | + | 0.77 | 0.61 | 130.69 |
| bc |  |  | + | + | + |  |  |  |  |  | 0.21 | 0 | 144.26 |
| bd |  |  | + |  |  | + | + | + | + | + | 0.37 | 0.06 | 144.54 |
| cd |  |  |  | + | + | + | + | + | + | + | 0.43 | 0.05 | 144.97 |
| a | + | + |  |  |  |  |  |  |  |  | 0.14 | 0 | 143.53 |
| b |  |  | + |  |  |  |  |  |  |  | 0.07 | 0 | 142.83 |
| c |  |  |  | + | + |  |  |  |  |  | 0.18 | 0.05 | 142.76 |
| d |  |  |  |  |  | + | + | + | + | + | 0.33 | 0.09 | 143.49 |
| Final Model | + |  |  |  |  |  |  |  |  | + | 0.63 | 0.57 | 130.11 |

a: neuropsychological cluster.

b: vocal intensity cluster.

c: prosody cluster.

d: articulation cluster.
